# Downregulation of ATP binding cassette subfamily a member 10 acts as a prognostic factor associated with immune infiltration in breast cancer

**DOI:** 10.18632/aging.203933

**Published:** 2022-03-05

**Authors:** Pei-Yi Chu, Yen-Dun Tony Tzeng, Kuan-Hao Tsui, Ching-Yu Chu, Chia-Jung Li

**Affiliations:** 1 Department of Post-Baccalaureate Medicine, College of Medicine, National Chung Hsing University, Taichung 402, Taiwan; 2 School of Medicine, College of Medicine, Fu Jen Catholic University, New Taipei City 242, Taiwan; 3 Department of Pathology, Show Chwan Memorial Hospital, Changhua 500, Taiwan; 4 Department of Health Food, Chung Chou University of Science and Technology, Changhua 510, Taiwan; 5 National Institute of Cancer Research, National Health Research Institutes, Tainan 704, Taiwan; 6 Department of Surgery, Kaohsiung Veterans General Hospital, Kaohsiung 813, Taiwan; 7 Institute of Biomedical Sciences, National Sun Yat-sen University, Kaohsiung 804, Taiwan; 8 Department of Obstetrics and Gynecology, Kaohsiung Veterans General Hospital, Kaohsiung 813, Taiwan; 9 Institute of BioPharmaceutical Sciences, National Sun Yat-sen University, Kaohsiung 804, Taiwan

**Keywords:** breast cancer, ABCA10, prognosis, biomarker, multi-omics

## Abstract

The human ATP binding cassette (ABC) family of transporter proteins plays an important role in the maintenance of homeostasis *in vivo*. The aim of this study is to evaluate the potential diagnostic, prognostic, and therapeutic value of the ABCA10 gene in BRCA. We found that ABCA10 expression was downregulated in different subgroups of breast cancer and strongly correlated with pathological stage in BRCA patients. Low expression of ABCA10 was associated with BRCA patients showing shorter overall survival (OS). ABCA10 expression may be regulated by promoter methylation, copy number variation (CNV) and kinase, and is associated with immune infiltration. Our study also demonstrated the potential role of ABCA10 modifications in tumor microenvironment (TME) cellular infiltration. Nevertheless, the regulatory mechanism remains unknown and immunotherapy is marginal in BRCA. We demonstrate the expression of different ABCA10 modulators in breast cancer associated with genetic variants, deletions, tumor mutation burden (TMB) and TME. Mutations in ABCA10 are positively associated with different immune cells in six different immune databases and play an important role in immune cell infiltration in breast cancer. Overall, this study provides evidence that ABCA10 could become the potential targets for precision treatment and new biomarkers in the prognosis of breast cancer.

## INTRODUCTION

Breast cancer is one of the most common malignant tumors in women, with the highest incidence and mortality rate among all types of malignant tumors in women, posing a serious threat to women’s health [1]. In recent years, the treatment of breast cancer has been developing, and various clinical treatments such as surgery, chemotherapy and targeted therapy have become more and more mature. However, due to the high heterogeneity and metastatic nature of breast cancer, there is still room for improvement in the mortality rate of breast cancer patients [2, 3]. Therefore, it is important to select more effective drugs for breast cancer treatment, prolong the survival of patients and reduce the mortality rate of breast cancer.

The ATP-binding cassette transporter protein (ABC transporter protein) family is a class of transmembrane transport proteins widely found in living organisms [4]. The ABC transfer protein superfamily is the largest family of human transfer protein genes, which is divided into seven subfamilies: ABCA, ABCB, ABCC, ABCD, ABCE, ABCF, and ABCG, the results of which can be divided into full and half-transporter. To date, 48 ABC genes have been identified, most of which are membrane-bound primary transporter proteins that actively transport various molecules to all cell membranes via ATP hydrolysis. The typical structure of ABC transporter proteins consists of a pair of nucleotide-binding domains (NBDs) located on the cytoplasmic side of the membrane, where the NBDs function to bind and hydrolyze ATP to provide energy for substrate translocation and the transmembrane domains (TMDs) are involved in substrate recognition [5]. Many cancers have been associated with ABC transport protein mutations, including ovarian cancer, lung cancer, liver cancer, colorectal cancer, and leukemia [4, 6–10]. However, there are no reports of low expression and mutation of ABCA10 leading to the progression of breast cancer.

The use of bioinformatics to identify important cancer biomarkers is increasingly becoming a reliable and profitable method to provide a reliable guide for developing appropriate therapeutic interventions due to the availability of multi-omics clinical data in public databases including differentially expressed genes, mutation signatures, treatment response and survival characteristics of cancer patients. In addition, network analysis of multi-omics data also helps us to understand the epigenetic mechanisms of cancer development and facilitate the discovery of epigenetically based prognostic biomarkers and therapies. In this study, we identified ABCA10 as an oncogenic predictor of breast cancer and tumor immune infiltration. We also demonstrated that the ABCA10 signature was associated with immunotherapeutic response and poor prognosis in a breast cancer cohort. Genetic alterations in ABCA10 co-occur with other genetic alterations and are associated with poorer prognosis in the cohort. Finally, through pharmacogenetics we screened for drugs that have potential to target ABCA10. Thus, our findings may be clinically useful in designing appropriate treatment strategies, prognostic assessment and follow-up management of multiple cancer immunotherapies.

## MATERIALS AND METHODS

### Multiple breast cancer cell lines and cell culture

Normal breast cells (H-184B5F5/M10) and breast cancer cell lines (MDA-MB361, MDA-MB-231, MDA-MB-453, MDA-MB-468, HS578T, ZR781, T47D and MCF7 (all cell lines were purchased from (bioresource collection and research center, Hsinchu, Taiwan) were used and incubated in culture medium supplemented with 10% fetal bovine serum in a humidified atmosphere with 95% air and 5% CO_2_ at 37°C, while the MDA-MB cell line did not require CO_2_ conditions.

### Real-time PCR detection

Multiplex breast cancer cell lines were extracted using EasyPrep Total RNA Kit (BIOTOOLS Co., Ltd., Taipei, Taiwan). and reverse transcribed by ToolScript MMLV RT kit. (BIOTOOLS Co., Ltd.). RT-qPCR was performed using TOOLS 2X SYBR qPCR Mix (BIOTOOLS Co., Ltd.) in a StepOne™ Real-Time PCR System (Thermo Fisher Scientific).

### Human BRCA specimens

Tissue microarray (TMA) slides (CBA4) containing human breast cancer, metastatic, and normal tissues were purchased from SuperBioChips Laboratories (Seoul, Republic of Korea). For immunohistochemistry (IHC) assays and scoring methods were performed as described. The slides were treated with anti-ABCA10 antibody (1:100, Merck, USA). IHC analyses included a scoring system involving two aspects, namely, staining intensity and percentage of positive cells. The total score ranged from 0 to 300, calculated as staining intensity × percentage of positively labeled cells. All clinical studies were performed in accordance with the approved guidelines of the Show Chwan Memorial Hospital Institutions Review Board (IRB: 1080604). Informed consent was obtained from all patients involved in this study.

### cBioPortal database

cBioPortal (http://cis.hku.hk/TISIDB/) is a comprehensive website that allows exploration, visualization and analysis of multidimensional cancer genomic data [11]. We obtained the frequency of ABCA10 gene changes, mutation types and copy number changes in all tumors in TCGA through the "Cancer Type Summary" module of cBioPortal. In this study, we selected “TCGA Pan Cancer Atlas Study” in “Quick Select” section of the cBioPortal web and entered into “ABCA10” to find the genetic alteration characteristics of ABCA10. Next, we observed the alteration frequency results, structural variants, mutation type, and CNA (Copy number alteration) of all TCGA tumors within the “Cancer Types Summary” module.

### GEPIA 2 database

GEPIA2 (http://gepia2.cancer-pku.cn/#index) is a web-based interactive tool for analyzing relevant RNA sequencing data from the cancer TCGA and GTEx projects [12]. General gene expression profiling, survival analysis and correlation analysis of TCGA-BRCA cohorts and normal tissues are performed through the "Expression Analysis" module, and data are available in the panel "dataset sources" (setting: *P*-value cutoff = 0.01, log2 fold change cutoff = 1, and “Match TCGA normal and GTEx data”). Student *t* tests were used to perform expression analyses. Survival results are shown by Kaplan-Meier curves. *p* value = 0.05 is used as a threshold for statistical significance.

### Kaplan-meier plotter

For survival analysis in Kaplan-Meier Plotter (https://kmplot.com/analysis/), patient groups were divided by “Auto select best cutoff”, which automatically computes all possible cutoff values between the lower and upper quantile and selects the best performing threshold as a cutoff.

### LinkedOmics database

LinkedOmics (http://www.linkedomics.org/admin.php) is an interactive portal that includes 32 TCGA cancer-related data. Differentially expressed genes associated with ABCA10 in BRCA were analyzed using the Pearson test [13]. We used the LinkedOmics functional module to analyze co-expressed genes of ABCA10 to explore their biological significance in BRCA. We downloaded the TCGA dataset of breast cancer mRNA and screened 1093 clinical cases containing ABCA10 gene expression, and ranked the cases in the top 50% and bottom 50% of expression levels as the high and low expression groups, with a test standard of *p* < 0.001.

### Oncomine database

The Oncomine database was used to determine the transcriptional expression level of ABCA10 gene in breast cancer. The expression level of ABCA10 mRNA (log2 transactivation) in BRCA tissues was evaluated relative to its expression in normal tissues [14]. To obtain the most significant ABCA10 expression, the thresholds were set as follows: the *p*-value was set as 1E−4, and the fold change was set as 2.

### Breast cancer gene-expression miner 4.7

Evaluate the expression and prognostic value of ABCA10 in breast cancer using the Breast Cancer Gene Expression Miner online dataset [15]. The online dataset is a statistical mining tool for published annotated breast cancer transcriptomic data, including DNA microarray, RNA-seq, and RNA-seq. RNA-seq has a large amount of published annotated genomic data, allowing statistical analysis of gene expression, correlation and prognosis.

### TIMER 2.0 database

Tumor Immunology Estimation Resource (TIMER) 2.0 is providing a profile of various infiltrating immune cells (B cells, CD4+ T cells, CD8+ T cells, neutrophils, macrophages and dendritic cells...etc.) in tumor tissues as detected by RNA-Seq expression profiling data. We evaluated the association of ABCA10 expression levels with immune cell infiltration, survival of BRCA patients as derived from different databases in TIMER, and immune cell infiltration with genes. We entered "ABCA10" in the "Gene_DE" module of the TIMER 2.0 and found differences in ABCA10 expression between adjacent normal tissues and 33 different tumors or specific tumor subtypes in the TCGA project.

### Connectivity map analysis

To identify potential drugs capable of mimicking ABCA10 activation, differentially expressed genes (DEGs) from ABCA10 overexpressing MCF7 cells were prepared using the R-based web application GEO2R [11]. The cMAP database collects drug-induced gene expression profiles from human cancer cell lines and can be used to compare similarities and differences between the expression of the input DEGs and drug-induced genes.

### Statistical analyses

Statistical methods were as previously described [16]. Correlation of gene expression was assessed using Spearman’s correlation coefficient. Statistical differences were analyzed using GraphPad Prism (GraphPad Software, La Jolla, CA, USA) by performing a *t*-test or Fisher’s exact test for both groups and a one-way ANOVA test for one group. A *p*-value of less than 0.05 was considered statistically significant.

### Data availability statement

The dataset supporting the conclusions of this article is included within the article.

## RESULTS

### Identification of key mutated genes in BRCA

**Figure 1:**
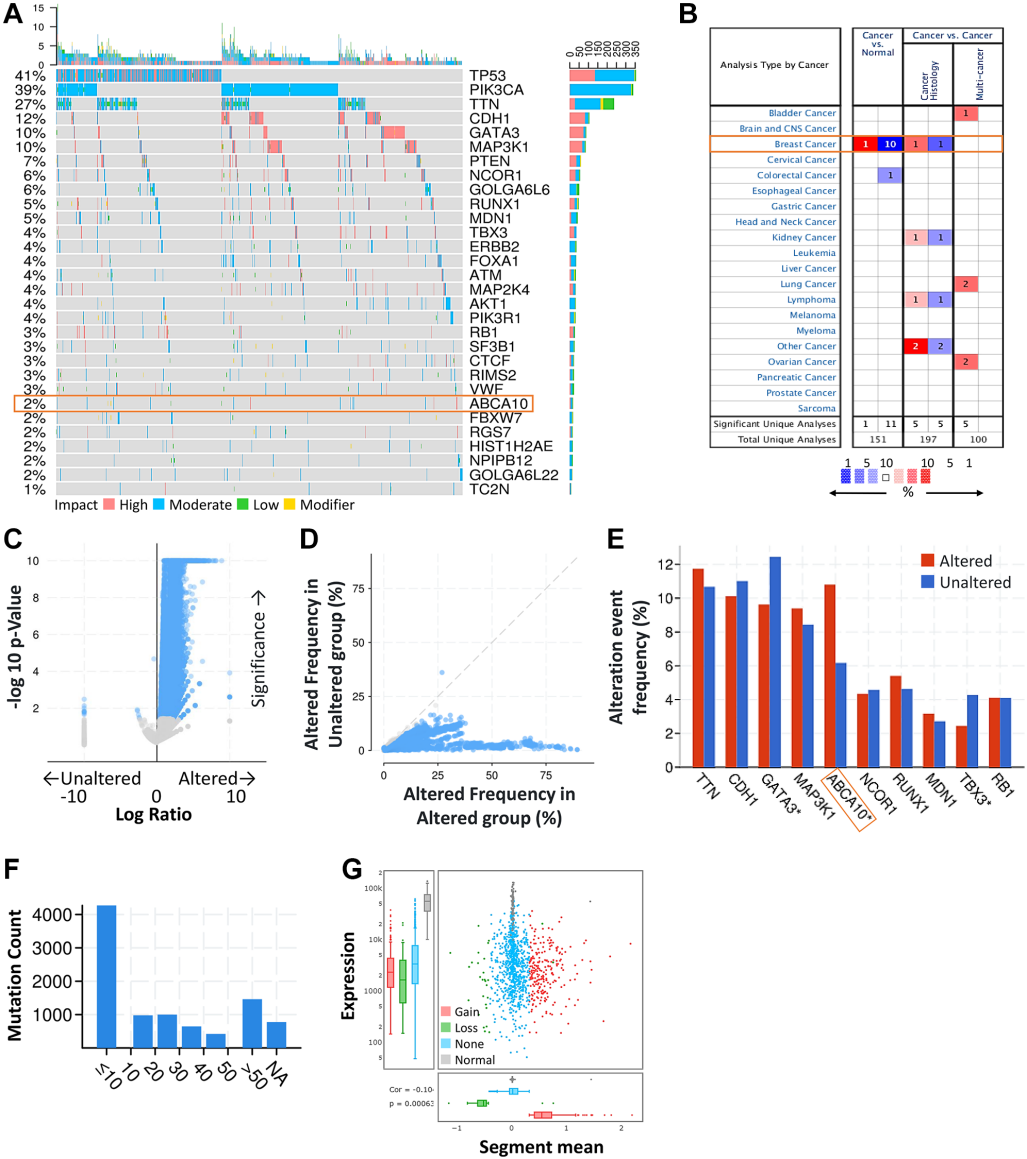
**ABCA10 was significantly mutated in breast cancer compared with normal breast tissue.** (**A**) Waterfall Plot of the top 30 mutated genes from TCGA. The bar plot indicates the number of genetic mutations per patient, while the right bar plot displays the number of genetic mutations per gene. (**B**) The mRNA expression levels of ABCA10 in multiple cancers on ONCOMINE database. Red background with numbers indicates the studies including ABCA10 expression levels meeting our selection standards (with *P*-values <0.05 and expression fold changes >1.5-fold change and expressed gene rank in the top 10% as our selection threshold) in cancer tissue; Blue (the same selection threshold) in normal tissues. (**C**, **D**) Volcano and scatter and volcano plots exhibiting genes associated with alterations in ABCA10 CNA frequency. (**E**) Box blot representing the 10 most frequently altered genes. (**F**) Mutation counts in patients with different kinds of cervical cancer in the TCGA dataset. (**G**) The distribution and correlation of CNVs are labeled as gains and losses, presented as visual ratios.

First, we obtained the mutation profiles of BRCA patients from the TCGA database. The details of the top 30 most frequently mutated genes are shown in the waterfall diagram as shown in Figure 1A. The validation of the TCGA-BRCA cohort indicates that ABCA10 is one of the frequently mutated genes. After analysis through the ONCOMINE database, we found that ABCA10 levels were much lower than normal tissues only in breast cancer among the pan-cancers (Figure 1B). Analysis of the frequency of concurrent gene alterations with ABCA10 gene alterations by the cBioPortal database revealed a total of 9083 genes with concurrent gene alterations, which were enriched for both ABCA10 altered and unaltered cohorts (Figure 1C, 1D). However, TTN, CDH1, GATA3, MAP3K1, ABCA10, NCOR1, RUNX1, MDN1 TBX3, RB1 altered and non-altered were the most common mutation cohort of genes altered (Figure 1E). We assessed the mutation load of each type of breast cancer by counting the mutations in each tumor sample. Most breast tumors had a mutation load in the <10 change range (Figure 1F). Significant changes in ABCA10 gain and loss were observed in the CNV ratio distribution and box plot (Figure 1G).

### Basic characteristics and genetic alteration of ABCA10 in BRCA

**Figure 2:**
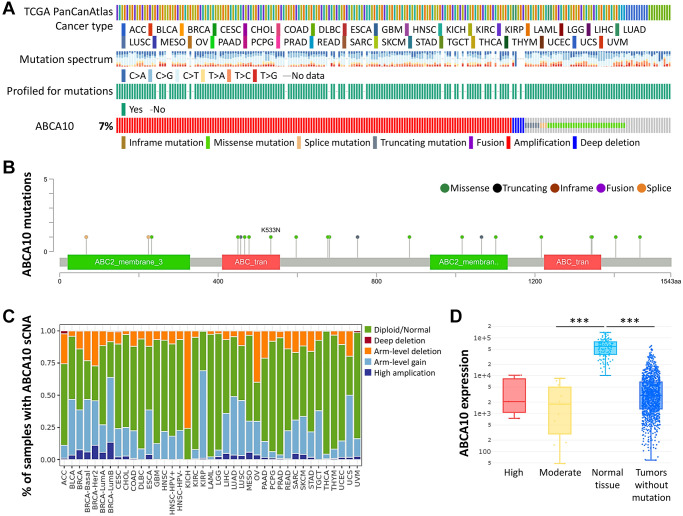
**Frequency and type of ABCA10 alterations in breast cancer.** (**A**) Analysis of various mutations in the ABCA10 gene in human cancer data. (**B**) The graphical view showing the ABCA10 protein domain and the location of specific mutations. (**C**) The illustration of the definition of somatic cell copy alteration in ABCA10 deletion, arm and chromosome levels. (**D**) Expression of ABCA10 in different types of mutated tumor tissues.

Next, we used the cBioPortal database to evaluate the type and frequency of ABCA10 alterations in BRCA tissues based on sequencing data from BRCA patients obtained from TCGA’s Pan-Cancer Atlas database. We found that 7% of ABCA10 genes were mutated in various cancers (Figure 2A). We further explored the specific alterations in each gene, and we also found that residues 400–500 had the most mutated sites in the ABCA10 structure. All genetic alterations occurring in BRCA tumor samples were mostly copy number amplification (Figure 2B), which was the predominant type of genetic alteration in all TCGA tumor samples. The somatic copy number alterations (sCNA) module allows the user to compare the immune infiltration distribution of TCGA cancer types by the sCNA status of the genes. We examined the expression of many representative genes from each of the major ABCA10 pathways and investigated ABCA10 “deep deletion” or “high amplification” altered states. We observed the gene expression levels of ABCA10 master regulators in pan-cancer (Figure 2C). In this study, we present an analytical strategy to assess the relative prognostic impact of all arm-level events in a pan-cancer SCNA cohort by varying mutation percentages in the pan-cancer SCNA cohort. This combined landscape of arm-level gains highlights the specific changes most closely associated with survival within human breast cancer and each specific type. Importantly, this complete list of rankings represents several attractive candidate gene sets and functions to explore. Next, we investigated the relationship between ABCA10 expression and BRCA mutation type. The results showed significant differences between the moderate and normal tissue and tumors without mutation groups (Figure 2D).

### Downregulation of ABCA10 signal network is associated with poor prognosis

**Figure 3:**
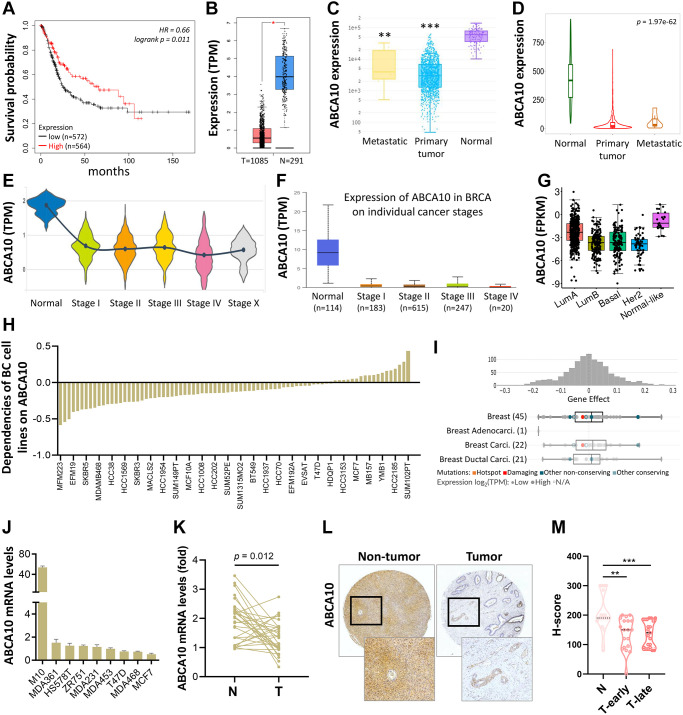
**Transcriptional level of ABCA10 in BRCA.** (**A**) Overall survival estimates for ABCA10 mRNA levels from Kaplan-Meier plotter database. (**B**) Expression of ABCA10 in BRCA and normal tissues. (**C**, **D**) ABCA10 expression in normal, BRCA primary tumor and metastatic tumor from different datasets. Violin (**E**) and box plot (**F**) to evaluate ABCA10 mRNA expression in BRCA patients based on pathological stage. (**G**) The mRNA expression level of ABCA10 among different subtypes of BC from TCGA database. (**H**, **I**) Significance of dependency of ABCA10 in 84 BRCA cell lines and different subtypes based on the CRISPR screen. (**J**) mRNA expression of ABCA10 in normal breast cells and multiple breast cancer cells. (**K**) qPCR analysis of ABCA10 in 30 paired BRCA and non-tumor tissues. N and T represent non-tumor and tumor tissues, respectively. (**L**) Representative images of ABCA10 staining in BRCA tissues. (**M**) IHC scores of ABCA10 expression in BRCA tissues. ^**^P < 0.01, ^***^P < 0.001.

**Figure 4:**
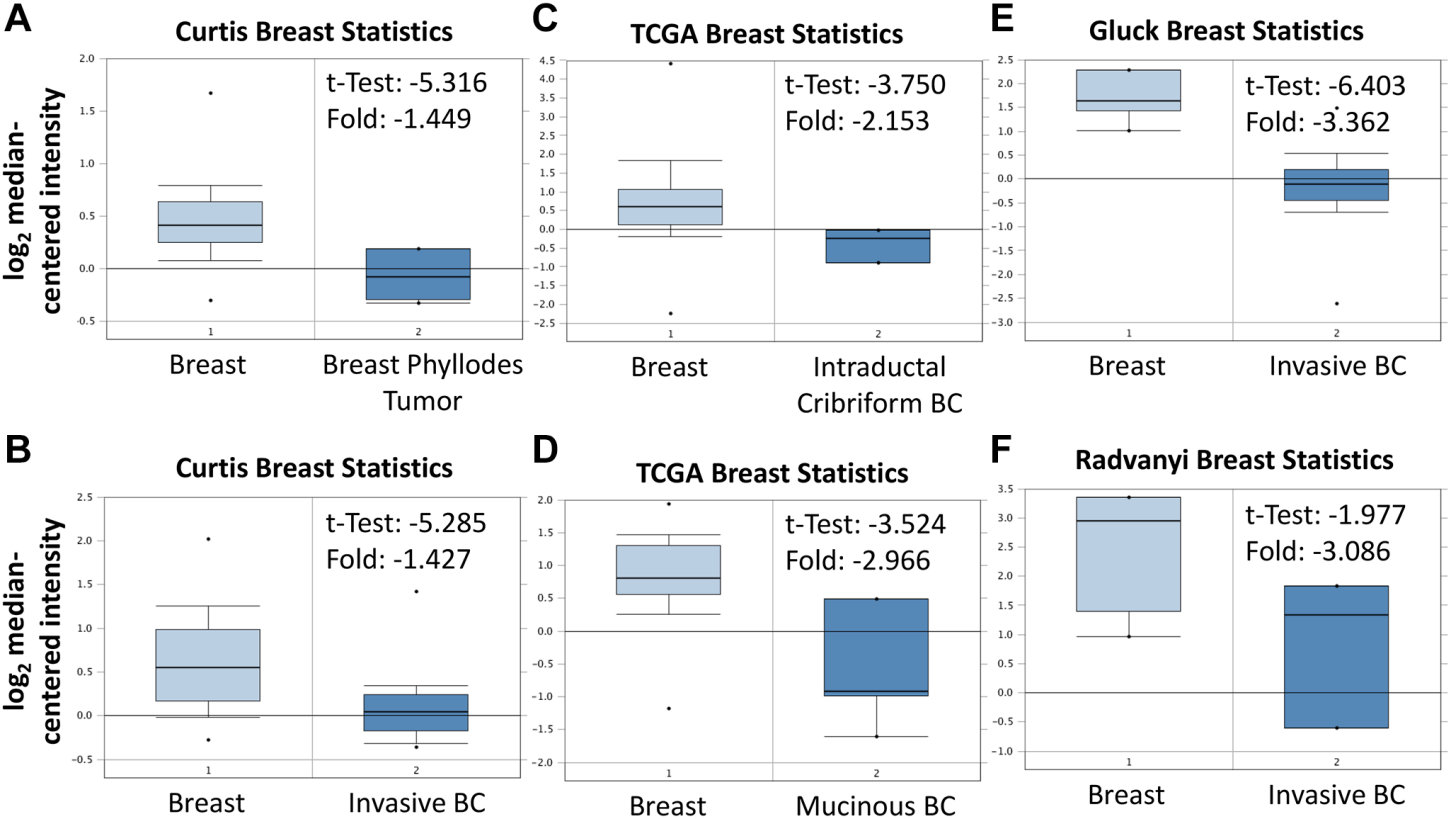
**Expression of ABCA10 gene in breast cancer in Oncomine database.** ABCA10 mRNA levels from (**A** and **B**) Curtis Breast statistics cohort, (**C** and **D**) TCGA Breast Statistics cohort, (**E**) Gluck Breast Statistics cohort (**F**) Radvanyi Breast Statistics cohort in BRCA and normal tissue. Note: *p* < 0.05 indicates statistical significance; ABCA10 was among the top 10% overexpressed genes in all four different datasets of BRCA.

As shown in Figure 3A, BRCA patients were divided into low ABCA10 and high ABCA10 groups using the median expression as the threshold, and the expression difference between the two groups was significant (*p* = 0.011). Kaplan-Meier analysis showed that the survival rate of the low-ABCA10 group was shorter than that of the high-ABCA10 group. We analyzed the expression levels of ABCA10 in normal and tumor tissues of the breast and showed a significant decrease in ABCA10 expression in tumor tissues (Figure 3B). The expression of ABCA10 in malignant breast cancer was shown by various databases, and the results showed that metastatic tissues were significantly lower than normal tissues (Figure 3C, 3D). To understand the levels of ABCA10 mRNA at different clinical stages, the data consistently showed that the levels of ABCA10 were significantly reduced from stage I (Figure 3E, 3F). The expression levels of ABCA10 in luminal A, luminal B, basal-like, and HER2 were all lower than normal tissues in different subtypes of breast cancer (Figure 3G). We further analyzed the dependence of 84 breast cancer cell lines on ABCA10 and mapped the ABCA10 dependence (fold change in sgRNA abundance relative to control transfected cells) of breast cancer cell lines and different subtypes, which were ranked by increasing ABCA10 dependence (Figure 3H, 3I). We further confirmed the mRNA levels of ABCA10 in breast cancer cells and normal breast cells (H-184B5F5/M10), and the results were consistent with the database data, where ABCA10 levels were significantly higher in normal breast cells than in other breast cancer cells (Figure 3J). Next, we examined the mRNA expression of ABCA10 in 30 paired BRCA and non-tumor tissues. The qPCR results showed that ABCA10 was significantly up-regulated in BRCA tissues (Figure 3K). To further confirm the accuracy of the multi-omics analysis, we evaluated ABCA10 detected using immunohistochemistry in tumor tissues using 60 BRCA commercial tissue microarray (TMA). The results of ABCA10 expression in BRCA tissues in IHC staining are shown in Figure 3L. The IHC score of ABCA10 decreased significantly from early stages and decreased significantly with the increase of late stages (Figure 3M). The results were consistent with the results of the Oncomine database, and where lower ABCA10 expression levels occurred at an early stage. Using the Oncomine dataset, we analyzed the levels of ABCA10 in normal breast tissue, breast phyllodes tumor, Intraductal Cribriform breast cancer, mucinous breast cancer, Invasive breast cancer, and other breast cancers. breast cancer. The expression of ABCA10 mRNA in BRCA was significantly lower than normal samples from all four datasets, and the sample size and fold changes corresponding to the four studies are summarized in Figure 4.

### ABCA10 expression levels in the subgroups of BRCA patients

**Figure 5:**
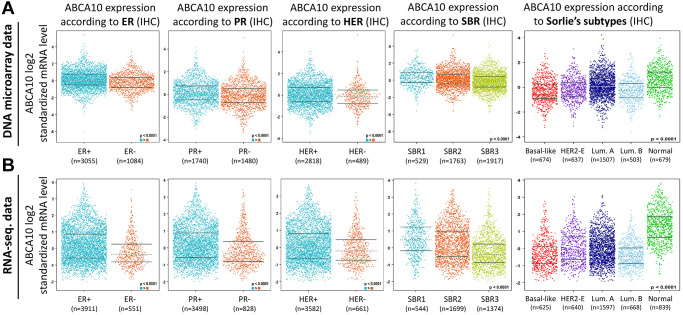
**Association between ABCA10 gene expression and clinical pathological parameters in patients with breast cancer.** (**A**, **B**) ABCA10 mRNA expression levels were shown in breast cancer patients by bee swarm in DNA microarray datasets and RNA-sequencing datasets. (Abbreviations: ER: estrogen receptor; PR: progesterone receptor; HER2: human epidermal growth factor receptor 2).

Both DNA microarray (Figure 5A) and RNA sequencing data (Figure 5B) confirmed consistent results for ABCA10 levels in ER+, PR+, HER+, (ER+ > ER-, PR+ > PR-, HER+ > HER-; *p* < 0.0001) with high expression. Analysis according to the Scarff Bloom and Richardson equivalence state (SBR) criterion showed that increased SBR levels correlated significantly with decreased ABCA10 levels (SBR1 > SBR2 > SBR3, *p* < 0.0001) in both DNA microarray and RNA sequencing data. Subsequently, results in different breast cancer subtypes also showed higher expression of ABCA10 in normal tissues than in other subtypes. Taken together, these DNA and RNA results provide prognostic value for the clinicopathological parameters of breast cancer.

### Functional network analysis of the predictive ABCA10 gene

**Figure 6:**
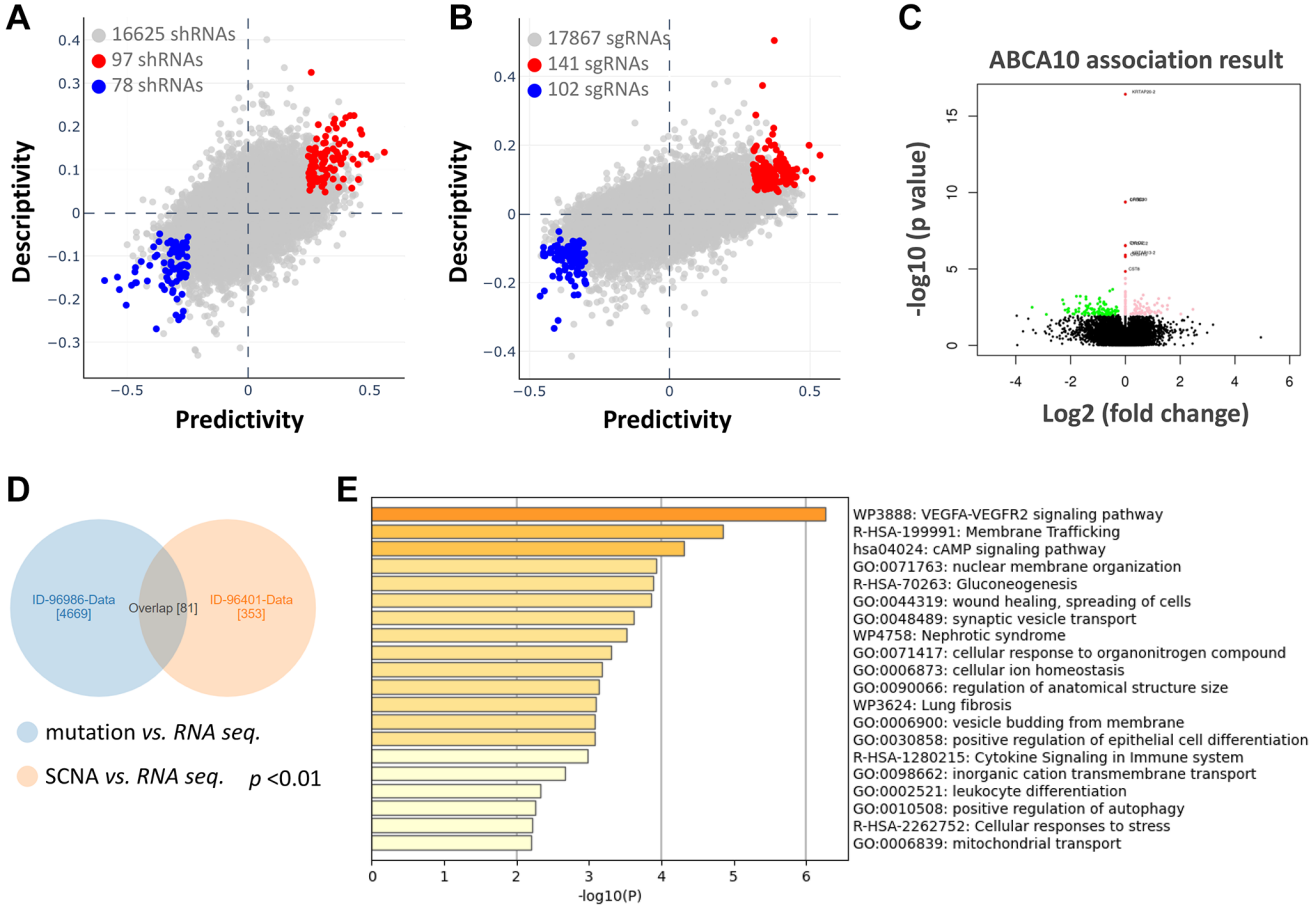
**Functional prediction and enrichment analysis of ABCA10 expression in breast cancer.** The predictability and descriptiveness between mRNA expression and shRNA (**A**) and sgRNA (**B**) functions are plotted with breast cancer cell lines. (**C**) Genes with shRNA/sgRNA overlap are identified in the positive correlation and negative correlation Venn diagram analysis. (**D**) Pearson test was used to analyze the differential gene expression related to ABCA10 in BRCA. (**E**) The top 20 functions of ABCA10 in BRCA are used for enrichment analysis.

We further analyzed the association between shRNA/sgRNA efficacy and the expression levels of target genes in different breast cancer cell lines. We attempted to calculate two-way predictive and descriptive scores for each of the more than 16,000-17,000 genes using statistical tests (Figure 6A, 6B). Among the shRNA efficacy, 97 genes (red circles in Figure 6A) showed positive scores in predictiveness and descriptiveness, while 78 genes (blue circles in Figure 6A) showed negative scores. Similarly, among the sgRNA efficacy, 141 genes (red circles in Figure 6B) showed positive scores in terms of predictiveness and descriptiveness, while 102 genes (blue circles in Figure 6B) showed negative scores. To further explore the potential functions and molecular pathways of ABCA10 genes in BRCA, we identified ABCA10 co-expressed genes in the data of 975 patients from TCGA using the LinkedOmics database. A total of 7,966 ABCA10-associated genes were altered, reflecting the important impact of the core gene ABCA10 on the pathogenesis of BRCA. These clusters of genes positively associated with ABCA10 are shown as red dots, whereas the clusters of genes negatively associated with ABCA10 are indicated by green dots in the volcano plot (*p* < 0.01 and FDR <0.01, Figure 6C). The 80 overlapping genes were analyzed by combining the two databases, and the top 20 significant gene clusters associated with ABCA10 were shown by functional enrichment (Figure 6D, 6E).

### Relationship between ABCA10 expression and BRCA-infiltrating immune cells

**Figure 7:**
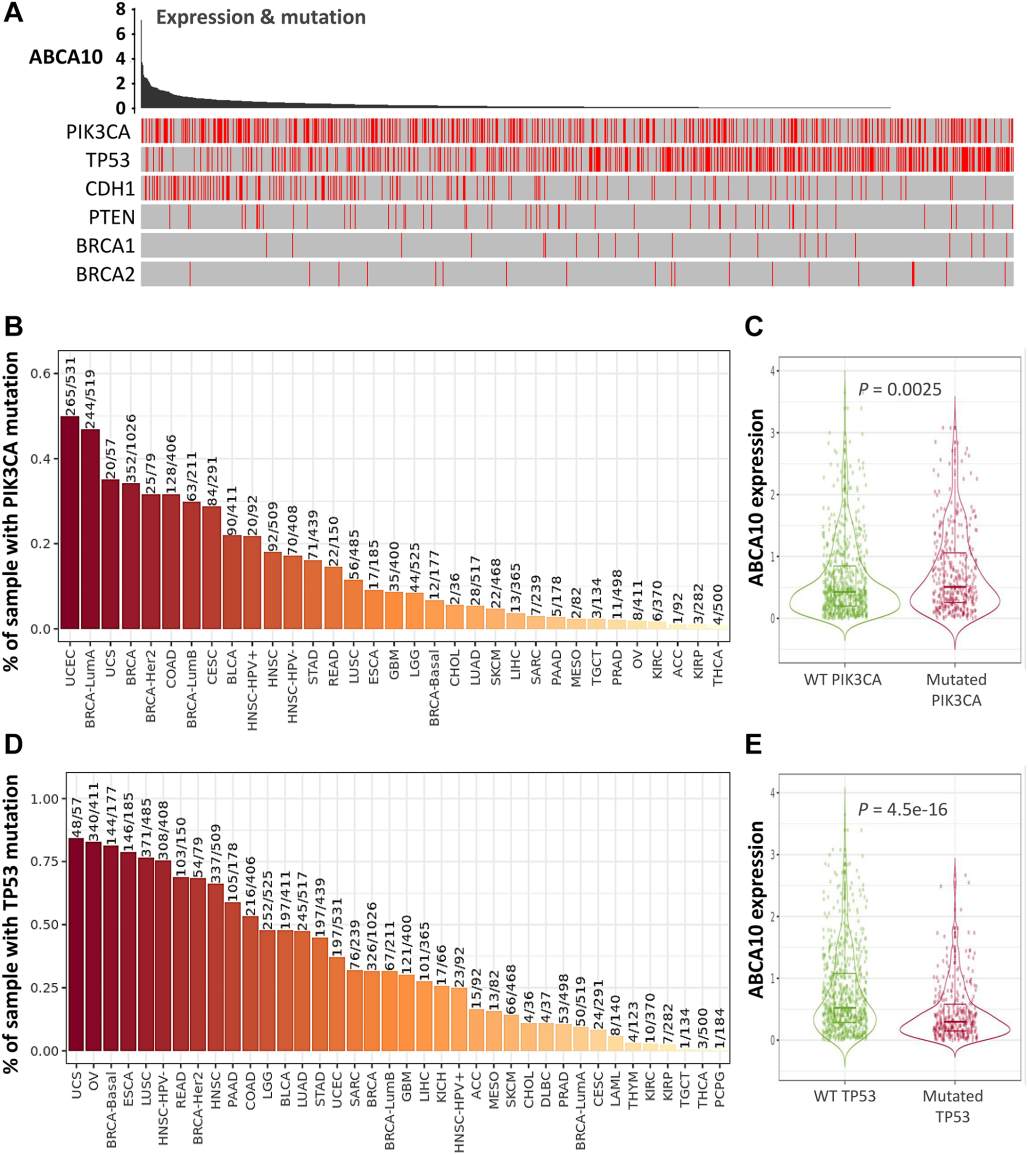
**Expression of common mutated genes and ABCA10 in BRCA.** (**A**) Relationship between ABCA10 and the six highly mutated genes in breast cancer. (**B**) Gene_Mutation module comparing PIK3CA mutation status among ABCA10 gene expression in pan-cancer. (**C**) Statistics of PIK3CA mutation status among ABCA10 gene expression in breast cancer (*n* = 1017). (**D**) Gene_Mutation module comparing TP53 mutation status among ABCA10 gene expression in pan-cancer. (**E**) Statistics of TP53 mutation status among ABCA10 gene expression in breast cancer (*n* = 1017).

**Figure 8:**
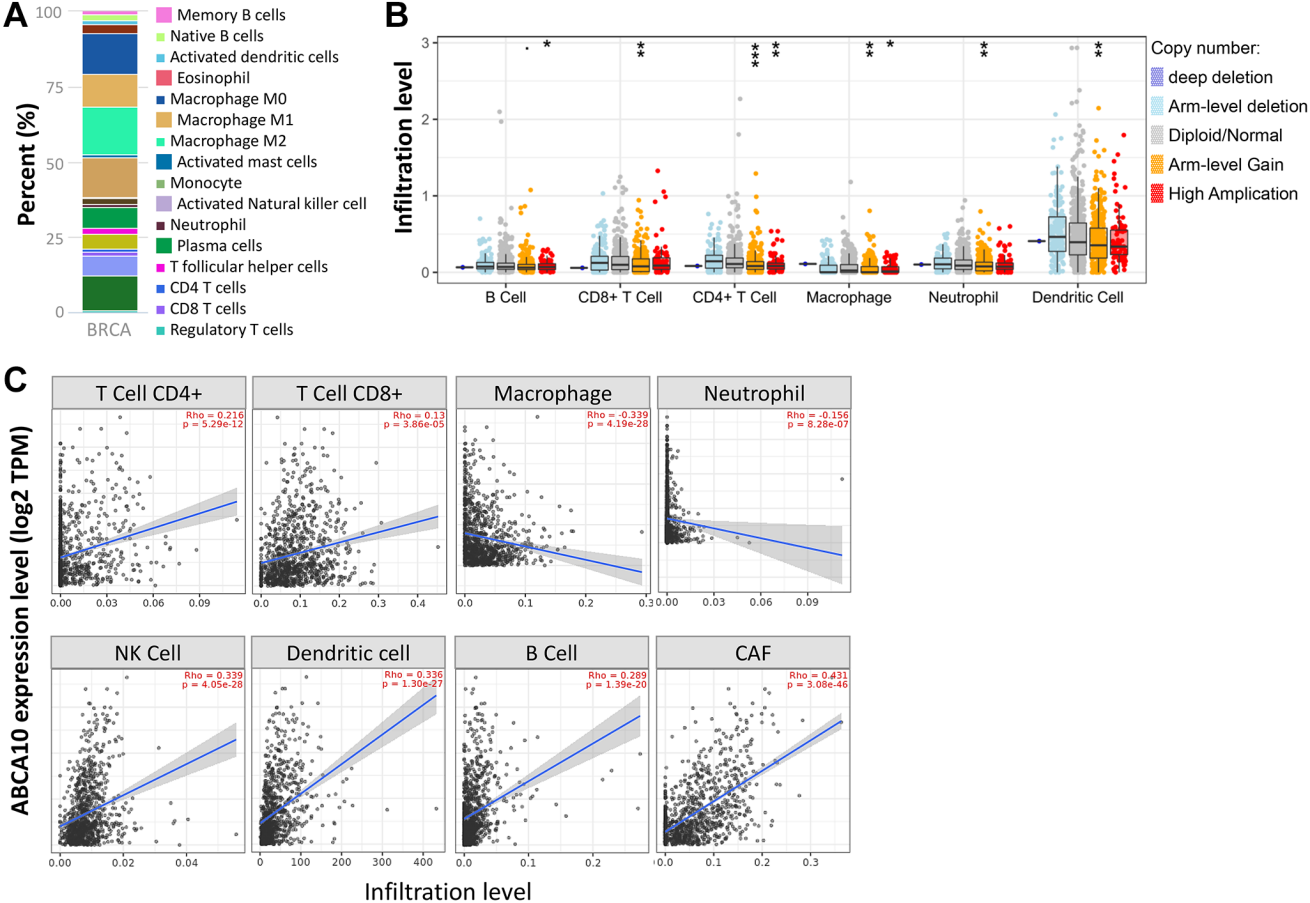
**Correlation of ABCA10 expression with immune infiltration level in BRCA.** (**A**) Immune cell bars show the expression of the ABCA10 gene. (**B**) The infiltration level of various immune cells under different copy numbers of ABCA10 in BRCA. (**C**) The correlation between ABCA10 expression level and immune infiltration. ^*^P < 0.05, ^**^P < 0.01, ^***^P < 0.001.

**Figure 9:**
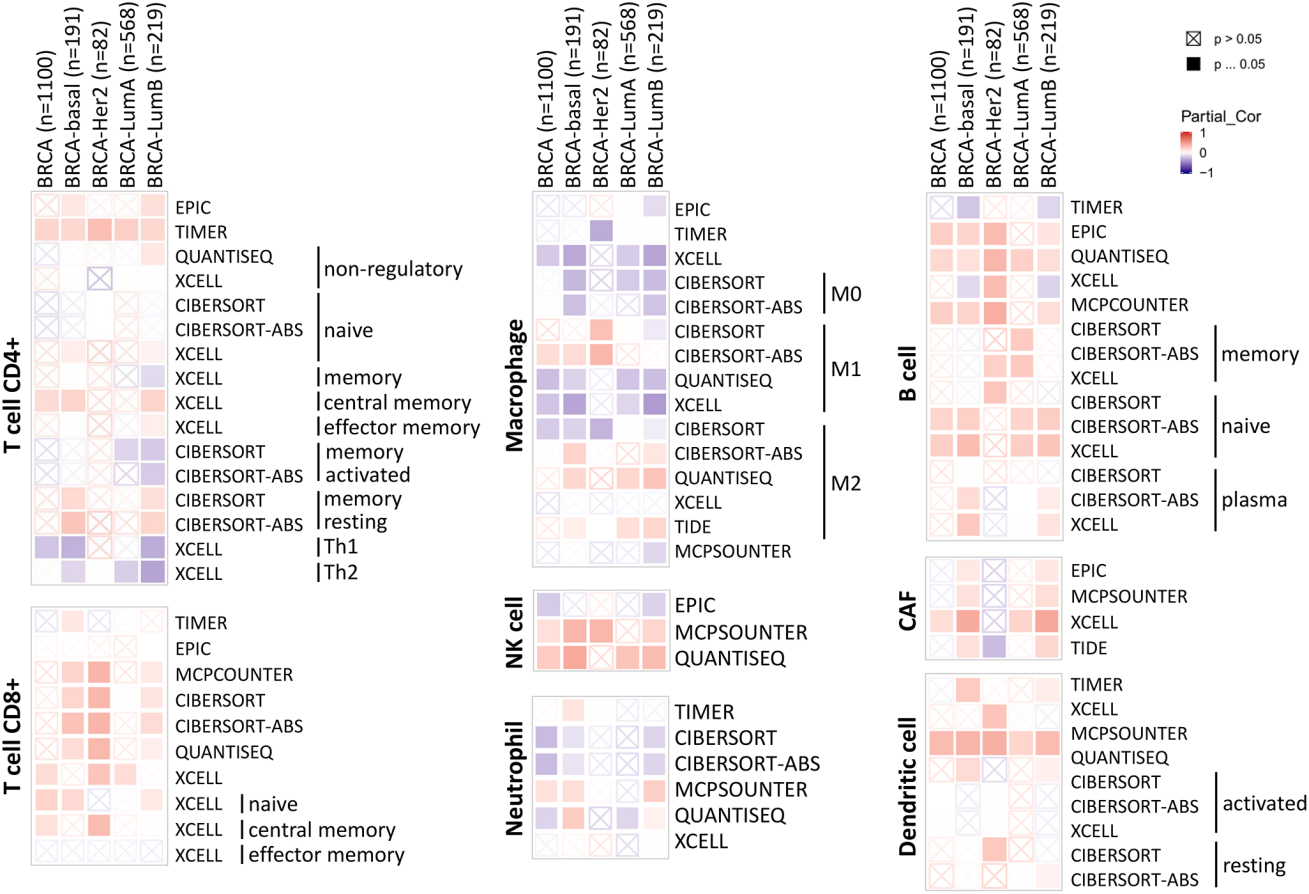
Correlation of ABCA10 expression with immune infiltration level in BRCA.

The tumor microenvironment is a highly complex system composed of multiple cells, enzymes, cytokines and metabolites, characterized by low oxygen, low pH and high pressure. To comprehensively investigate the role of ABCA10 in BRCA, we selected the TIMER database to analyze the association of ABCA10 expression levels with subpopulations of infiltrating immune cells. We evaluated the potential correlation between ABCA10 expression in breast cancer and several mutations commonly seen in breast cancer (Figure 7A). We found that the value of highly mutated genes adjacent to each other is driving the genetic variation distribution between mutated (red) and non-mutated (gray) samples. Then the effect of ABCA10 mutations on immune cell infiltration in various cancer types was analyzed by mutation modules in Pan-cancer. We analyzed the effect of ABCA10 mutations on immune cell infiltration by pan-cancer type and the effect of immune cell type on pan-cancer by mutation module (Figure 7B, 7D). The results showed that ABCA10 expression was significantly elevated in mutated PIK3CA (*p* = 0.0025) (Figure 7C). In contrast, ABCA10 expression was significantly decreased in mutant TP53 (Figure 7D). This also suggests that the association of ABCA10 with immunity may be related to the PIK3CA and TP53 mutations in BRCA (Figure 7E). In Figure 8A, ABCA10 is shown to modulate different immune cells in breast cancer cells, with macrophages M0, M1, M2 and Monocyte accounting for the highest percentage of immune cells. In addition, we further investigated the association between the CNV of ABCA10 and immune cell infiltration in the prognostic model. We found that deletion or amplification of other forms of ABCA10 compared to normal copy number may differentially modulate immune cell infiltration in breast cancer (Figure 8B). We further analyzed ABCA10 with various immune cells, and we found a positive correlation with T cells, NK cells, DCs, B cells, CAF; and a negative correlation with Macrophage and Neutrophil (Figure 8C). The relationship between ABCA10 and various tumor-infiltrating immune cells was evaluated by different immune databases. The results showed a significant correlation between ABCA10 and different levels of immune cell infiltration. Notably, ABCA10 expression showed a high positive correlation with the infiltration levels of CD8+ T cells, CD4+ T cells, B cells, CAF, DC, and NK cells; similar to the previous results, ABCA10 showed a negative correlation with Macrophage (Figure 9). Therefore, we hypothesize that the immune microenvironment plays a critical role in the development of breast cancer tumors and in the regulation of ABCA10.

### BRCA cells with lower ABCA10 expression are sensitive to the vasopressin receptor agonist

**Figure 10:**
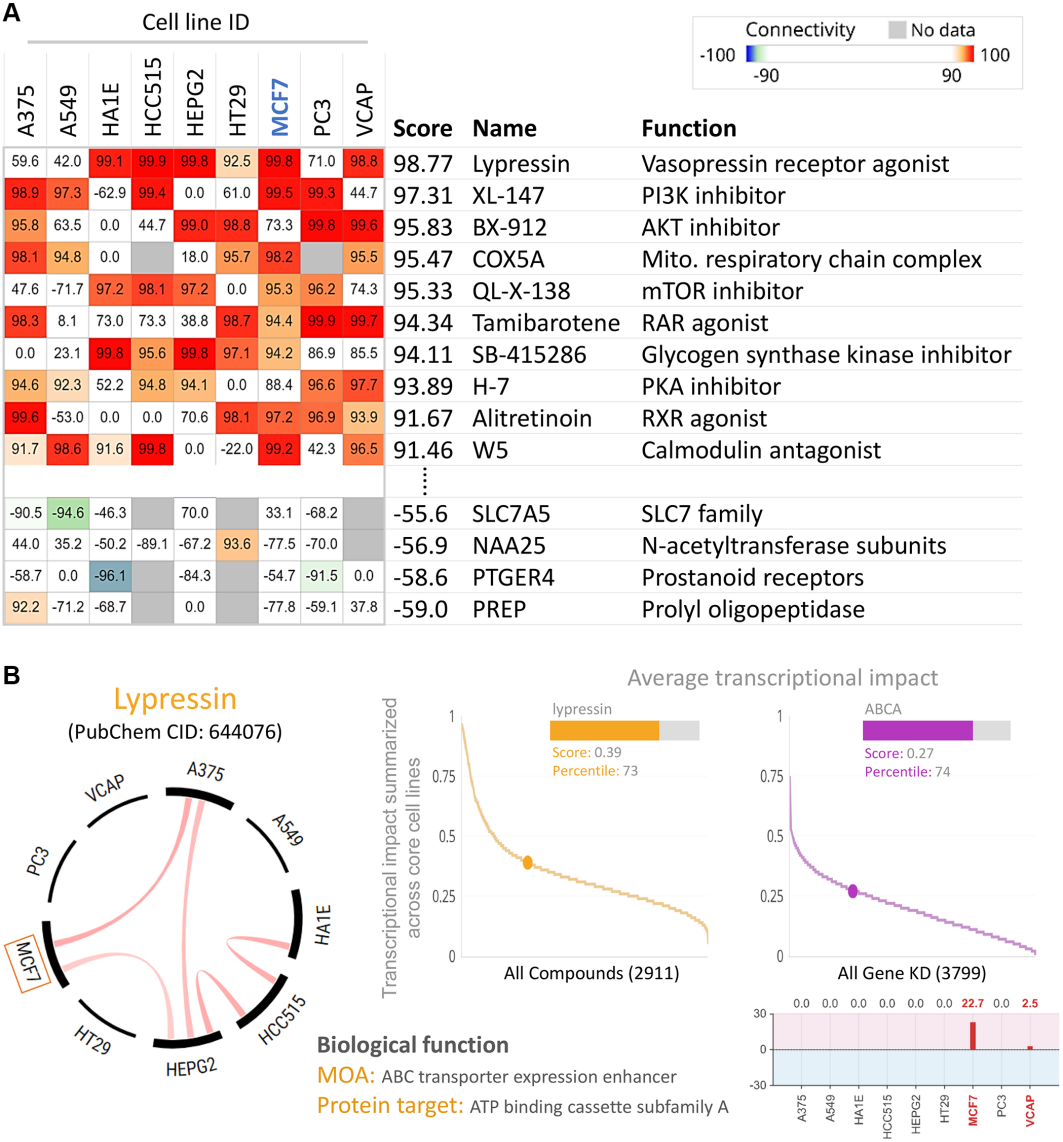
**Inhibition of ABCA10 expression in breast cancer cells by pharmacogenomic mapping.** (**A**) Lypressin treatment simulated the effects of ABCA10 inhibition on breast cancer cell lines. (**B**) Analyses were performed to explore the similarity between ABCA10 and drug-induced genetic characteristics in multiple cancer cell lines to assess the effects.

To find potential drugs targeted at ABCA10, we used connectivity Map (CMap) analysis. The CMap database provides gene signatures and filters for associations between specificity and drug-driven gene expression. The heatmap in Figure 10A shows the top 10 perturbants that mimic the ABCA10-driven gene signature, including Lypressin with a score of 98.77 as a Vasopressin receptor agonist, which regulates ABCA10 in MCF7 cells. In contrast, CAY-40145 was also a regulator of ABCA10 in MCF7 cells. As shown in Figure 10B, we found that the scores of Lypressin and ABCA10 KD on breast cancer cells (MCF7) were 0.39 and 0.27, respectively, indicating a positive correlation between the average transcriptional effect of ABCA10 expression and Lypressin drug activity. Thus, Lypressin treatment could mimic the gene expression profile of ABCA10 restoration.

## DISCUSSION

The occurrence, progression and impact on patient prognosis of breast cancer are closely related to abnormal intracellular signaling [3, 17]. These signaling pathways related to biological processes such as regulation of immunity, inhibition of tumor cell apoptosis, and tumor microenvironment are key links affecting tumor progression, and different signaling pathways are intertwined into a network and influenced by multiple targets [18, 19]. We found a high rate of ABCA10 gene amplification in pan-cancer, however, gene amplification is an increase in the difference between a specific part of the genome compared to the rest of the genome. This process seems to occur everywhere, in most organisms, and has also been shown to occur in germ cells and somatic cells. Amplification of proto-oncogenes causes or promotes tumorigenesis and/or tumor progression. Interestingly, the rate of missense mutation is also high, probably because some genes are missense leading to a high rate of amplification. In some cases, the amplified gene may be a useful target for cancer therapy. Immune cells in the tumor microenvironment are key elements of tumor tissue, and there is growing evidence to support their clinicopathological relevance in predicting survival status and treatment outcome in tumor patients [20, 21]. Specifically, the level of tumor-associated macrophages (TAM) infiltration accelerates cancer progression [22]. After controlling for confounding clinical features, multivariate analyses suggest that immune scores remain an independent prognostic factor, which was further validated in an independent cohort [23, 24]. These data suggest that immune scores have similar predictive power to traditional predictors [25]. TAM is composed mainly of M2 macrophages, possibly due to exposure to complex factors in the tumor microenvironment [26]. Figures 8, 9 shows that ABCA10 is closely associated with immune-related pathways, including B cell, CD8+ T cell, CD4+ T cell, Macrophage, neutrophil, and dendritic cell activation. By analyzing the relationship between ABCA10 and immune cell infiltration, only macrophage and neutrophil were negatively correlated, while other immune cells were currently correlated. Furthermore, the correlation between ABCA10 and immunosuppressive gene expression suggests that ABCA10 plays a key role in regulating tumor immunology. We have used bioinformatic analysis of the TCGA pan-cancer dataset to show that only breast cancer has significantly lower ABCA10 levels than normal tissue. We believe that genes with tumor suppressive functions that are repressed during tumorigenesis should at least be expressed in the corresponding normal tissues. Strikingly, only 20 of the 84 breast cancer cell lines were dependent on ABCA10 levels, suggesting that both clinical patients and breast cancer cells are sufficient to show that ABCA10 levels are low in the development of breast cancer. These findings provide strong evidence for a novel tumor suppressor function of ABCA10 in breast cancer. It is worth mentioning that the expression of ABCA10 has decreased significantly from the early stage, and the level of ABCA10 was the same in different tumor stages of BRCA.

Preventing tumor progression and suppressing tumor cells is an important task of the immune system, which involves not only T cells but also innate immune cells [5]. Notably, tumors are constantly developing strategies to reprogram the anti-tumor machinery in order to suppress the function of immune cells [27–29]. ABCB1 is an ABC transporter known for mediating multidrug resistance [30] and has been shown to regulate the memory function of CD8+ T cells [5]. ABC transporter is a modulatory tumor suppressor because it facilitates the execution of the cell death program through the mitochondrial pathway and achieves tumor suppression by regulating intracellular AKT signaling. The absence of the transport protein will affect the activation and expansion of CD8+ T cells, and will result in the accumulation of memory CD8+ T cells. Mechanistically, mitochondria are key regulators of ABC transport proteins through early activation and memory formation in CD8+ T cells [31]. The lack of ABC transporter protein increases the number of CD8+ and CD4+ T cells, accompanied by the production of IFN-γ activity by these cells [32]. Therefore, the identification of ABCA10 regulators may be a novel strategy to initiate the tumor environment to kill tumors.

There are some limitations in this study: all analyses were based on ABCA10 expression at DNA and mRNA levels, and conclusions were deduced from bioinformatics analysis, lacking more in-depth experimental data to support our mechanistic interpretation. Therefore, further studies are needed to validate our results and investigate the biologic function of ABCA10 in BRCA.

## CONCLUSIONS

The role of ABCA10 gene in breast cancer has not been reported. In this study, we observed the difference of ABCA10 gene expression in normal tissues and tumor tissues by means of big data analysis. The Kaplan-Meier prognostic survival curve analysis showed that the high expression of ABCA10 gene in breast cancer tissues indicates a good prognosis. This suggests that the detection of ABCA10 gene expression in breast cancer tissue has important clinical significance. We used bioinformatic prediction to initially screen out ABCA10, a gene differentially expressed between tumor and normal tissue, for further study and screening of potential drugs. Although we have tested the expression of ABCA10 in various breast cancer cell lines and confirmed the same as predicted, ABCA10 deserves further study to become a new breast cancer tumor marker.
